# Neurosurgical robot‐assistant stereoelectroencephalography system: Operability and accuracy

**DOI:** 10.1002/brb3.2347

**Published:** 2021-09-14

**Authors:** Di Zhang, Xuehua Cui, Jie Zheng, Shunyao Zhang, Meng Wang, Wenpeng Lu, Linxia Sang, Wenling Li

**Affiliations:** ^1^ Neurosurgery Department of Epilepsy The Second Hospital of Hebei Medical University Shijiazhuang China

**Keywords:** neurosurgery, robot‐assistant, stereoelectroencephalography

## Abstract

**Background:**

Fine operation has been an eternal topic in neurosurgery. There were many problems in functional neurosurgery field with high precision requirements. Our study aims to explore the operability, accuracy and postoperative effect of robot‐assisted stereoelectroencephalography (SEEG) in neurosurgery.

**Methods:**

We conducted a retrospective analysis of patients with epilepsy who underwent electrode implantation in our hospital. From 2016 to 2019, the epilepsy center of Hebei people's hospital implanted electrodes in neurosurgery on 24 patients, including 20 with SINO robot‐assisted SEEG system and eight with frame‐SEEG technology.

**Result:**

Robot‐assisted SEEG neurosurgery had higher accuracy, and the mean error of entry and target point was smaller than that of frame SEEG surgery. No bleeding or infection occurred postoperatively, and two patients who underwent robot‐assisted SEEG neurosurgery had electrode displacement. Electrode displacement was observed in two patients, both the entry points were orbital frontal, one in the frame system and one in the robot assistant system. The average placement time of each electrode in robot assisted system surgery was less than that in frame system surgery.

**Conclusion:**

The SINO SEEG electrode implantation assisted by surgical robot‐assistant system manufactured in China is safe, accurate and mature.

## Graphical abstract

Comprehensive epilepsy center is necessary to establish a multidisciplinary team of stereo EEG. SEEG electrode implantation assisted by surgical robot system is safe, accurate and mature. The close cooperation between engineers and clinicians, the continuous improvement of technical accessories and the constant update of software system have greatly promoted the development and application of domestic surgical robots. Intelligence and precision are the inevitable trend in the development of neurosurgery.

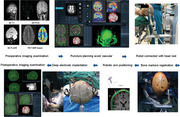

## 1 INTRODUCTION

Since the birth of modern neurosurgery in the late 19th century, fine operation has been an eternal topic in neurosurgery. In the past 20 years, technologies such as intraoperative imaging (Moriarty et al., 1996), brain functional imaging and neuron avigation (Mert et al., 2015; Morrison et al., 2016 ) have been regarded as landmarks of precision neurosurgery, but these technologies are more often applied to the subspecialty fields of neurosurgery, such as brain tumors and cerebrovascular diseases. The stereoelectroencephalograpy (SEEG) method was developed 50 years ago by Jean Bancaud and Jean Talairach at Sainte‐Anne Hospital in Paris, and they made the Talairach stereoscopic frame neurosurgical system. There are similar frame neurosurgical systems: Leksell and Fischer–Leibinger. SEEG is a kind of intracranial multi‐touch deep electrode placement and monitoring technology and is the best way to refractory epilepsy preoperative evaluation. It can better research anatomic‐electricity‐clinical seizures, epileptogenic zone in order to assist doctors better clear scope, achieve accurate resection and maximum protection in patients with brain function (Iida & Otsubo, 2017). The original Talairach frame‐based technology uses an external fixed grid system combined with intraoperative angiography and ventriculography in order to position the orthogonal electrodes in the desired position using a two‐dimensional image. Several epilepsy centers have described the technique of orthogonal and oblique depth electrode insertion into the insula by using the classic manual Leksell stereotactic framework, with good accuracy and few complications (Desai et al., 2011; Gil Robles et al., 2009; Salado et al., 2018 ). However, there were many problems in functional neurosurgery field with high precision requirements, such as how to realize the fusion of stereotactic technique and a variety of image data, how to achieve more accurate surgical plan and operation and how to realize the operation data real‐time feedback. It needed a new revolutionary technology and equipment to breakthrough development bottleneck, and the new frameless stereotactic surgery robot, Robot of Stereotactic Assistant (ROSA), arise at the historic moment (Lefranc et al., 2014). The frameless insertion technique adds a reference step before surgery, using a laser system to collect information about the patient's face and skull, to construct anatomic and radiological images of the patient, and to develop a surgical plan, simplifying the procedure and reducing time (De Barros et al., 2020). This technique was compared to frame‐based methods on SEEG in terms of complications but not accuracy (Abel et al., 2018).

**FIGURE 1:**
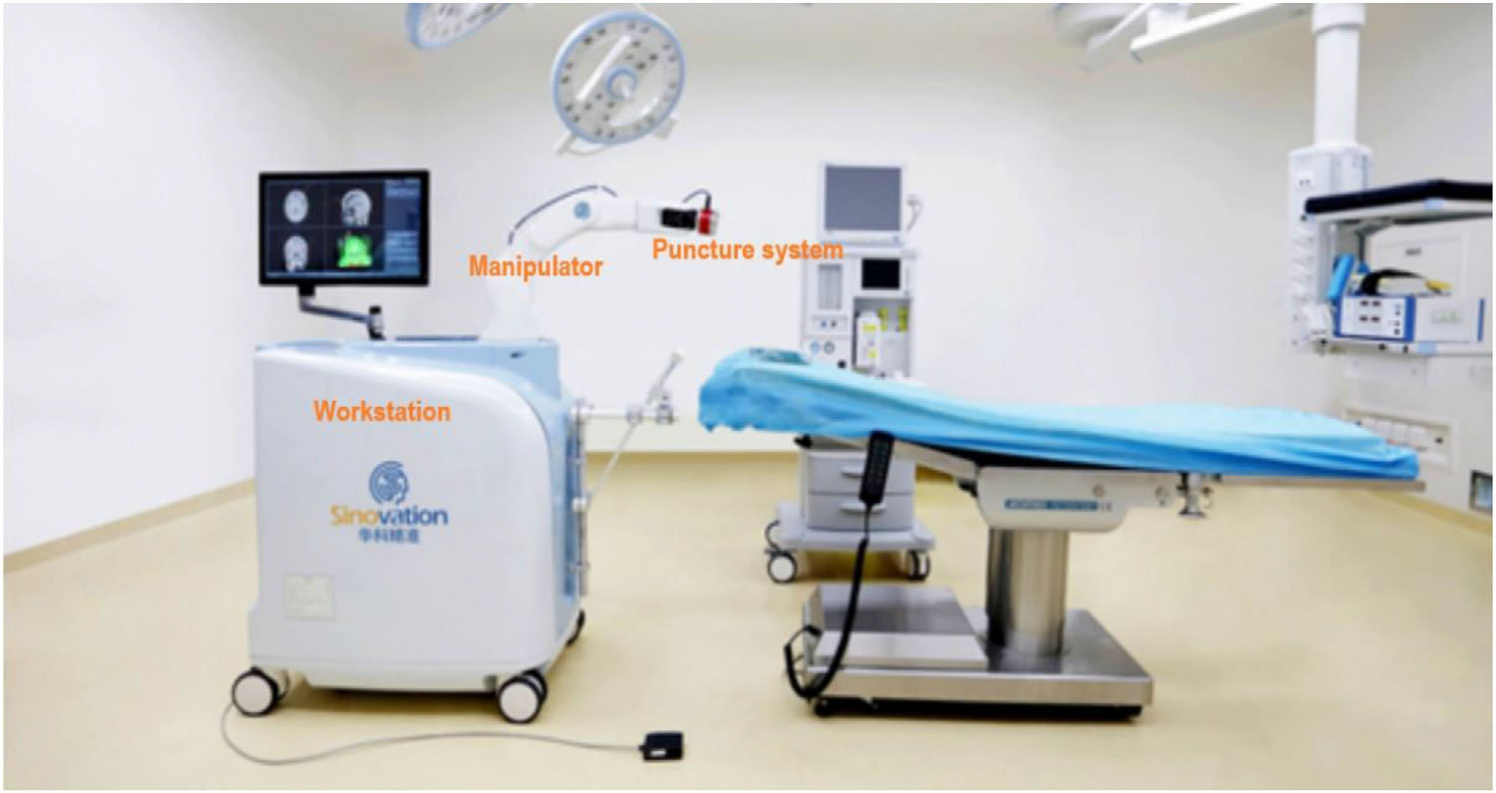
The SINO neurosurgical robot, including manipulator, puncture system, workstation and Sinoplan software system

In 2017, the SINO neurosurgical robot (Figure 1) produced by Sinovation Medical Technology Co., Ltd. (Beijing) was introduced into our hospital. SINO neurosurgical robot adopts the contactless visual location patient registration technology. Based on the way of robot arm ontology positioning, it adopts the method of automatic visual scanning to locate the patient's facial surface for patient registration. SINO neurosurgical robot has the three‐dimensional (3D) visualization technology of craniocerebral vascular based on multimodal image fusion, which can fully show the intracranial vascular structure. Combined with the results of radiological examination, a safer and more targeted puncture route and stereotactic surgical plan can be developed preoperatively. In this study, we analyzed the accuracy, complications and postoperative follow‐up results of operations to explore the advantages of robot‐assisted SEEG system applied on neurosurgery.

## 2 METHODS

### 2.1 Patients

From 2016 to 2019, the epilepsy center of Hebei people's hospital implanted electrodes in neurosurgery on 24 patients, including 20 with SINO robot‐assisted SEEG system and eight with frame‐SEEG technology. All patients included were diagnosed with moderate or severe epilepsy and required electrode implantation surgery. This study was approved by the Ethics Committee of The Second Hospital of Hebei Medical University (No. 2019‐R172). This was a retrospective study without the patient knowing.

### 2.2 Instruments and methods

SINO neurosurgical robot consists of hardware system and software system; hardware system includes manipulator, puncture system and workstation, and software system used is Sinoplan. The 6‐axis robotic arm system has the function of automatic touch avoidance, ensuring the dexterity and safety of the operation. The fixation mode of the robotic arm ensures accurate operation for physical and mechanical fixation. Sinoplan software system has a variety of functions, including multi‐mode image data (magnetic resonance imaging (MRI), computerized tomography (CT), vascular image and positron emission tomography (PET)) input and fusion, 3D reconstruction, vascular visualization, path planning and target calculation, data import robot hardware system assisted surgery and data output. The puncture planning system can carry out registration, fusion, 3D model extraction and path planning after importing preoperative image data. Puncture accessories include positioner, bit stepper, bit connecting rod, φ2.1 mm bit, electrocoagulation needle, probe, ruler and screwdriver.

**FIGURE 2:**
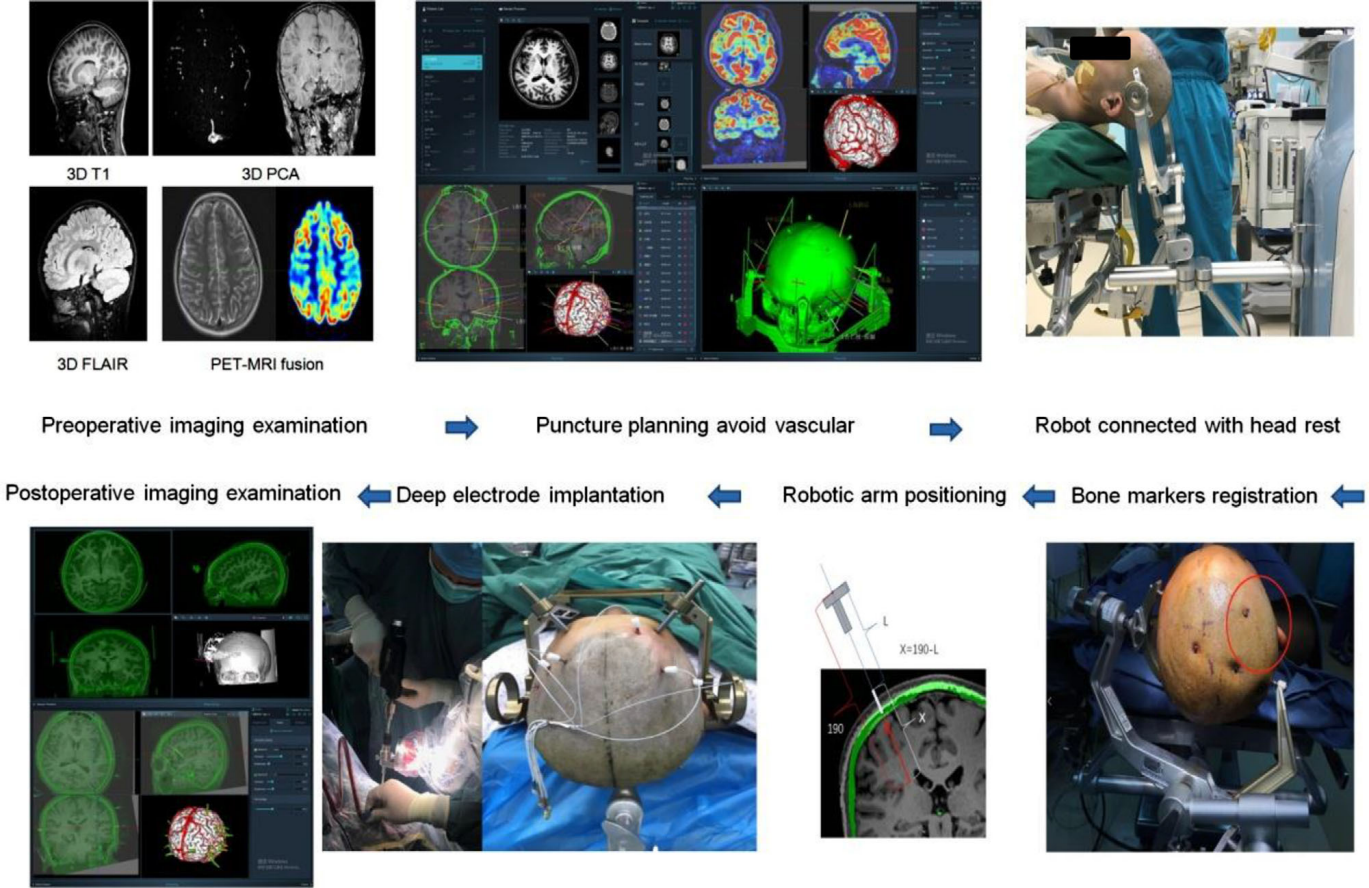
The simple procedure of SINO neurosurgical robot assisted stereoelectroencephalography (SEEG) electrode implantation

The procedure of SINO robot‐assistant SEEG electrode implantation is diagnosis, planning, implantation and postoperative. Diagnosis includes preoperative imaging examination, image data import, localization diagnosis or hypothesis of epileptic foci, based on electroencephalogram (EGG) results and symptomatology or other recordings, and 3D brain reconstruction, which was conducted to confirm the location, scope and interrelationship of lesions, epileptogenic foci and related important brain functional areas. Preoperative 3D structural and angiographic examination was performed. The anatomical structure can be observed with 3D‐T1. The lesions can be observed with 3D flair, and fat suppression should be set for 3D cortical reconstruction. 3D principal component analysis (PCA) can be used to observe magnetic resonance angiography without injecting contrast agent and without distinguishing between arteriovenous, and can be used for 3D vascular reconstruction. SINO software system supports dicom data import such as PET/CT, 3D digital subtraction angiography (DSA) and CT angiography (CTA). According to the implant data, the puncture planning system makes the path planning, identifies and avoids the vessels of the puncture path. Bone markers were installed on patient under local anesthesia, and the 3D‐CT scan for head was performed. CT data were fused with the implant plan to obtain the robot target path planning (6–15 items). Afterward, the patient went into the operating room for general anesthesia, and the head rest was fixed. The robot was connected to the head rest and fixed. For robot‐assistant SEEG surgery, the robot is typically placed directly in front of the patient's head. The linear distance between the base of the robotic arm and the patient's ear hole should be 50–55 cm, and the patient's head should be level with the base of the robotic arm. After the pendulum is completed, the robot will be locked by the lifting system. Use a headrest to secure the patient's head, and note that the rest should be away from the electrode covered area. Attach the robot rest to the other side of the rest, and then check and lock all joints. The data were output to the robot hardware system, and drag the robotic arm to the bone markers to complete the registration. After registration, the robotic arm will automatically be in place to perform the target puncture path. After the location of the markers is specified in the software, drag the robotic arm to successively point the probes at the bone markers to complete the registration. The registration accuracy of bone markers is about 0.1–0.4 mm. The robot automatically positioned itself to the planed path. Guided by the robot, the doctor drilled the skull with a 2.1 mm drill and a stepper, and then burned the dura mater with an electric coagulation needle. The robot was automatically positioned 190 mm away from the target, and the guide screw was installed with a screwdriver. Then, a 190 mm probe was used to make a puncture tunnel, and the distance from the end of the guide to the end of the guide screw was measured as L. Note that 190 mm minus L is the electrode depth X. Postoperative 3D‐CT scan was performed again, which was fused with preoperative head MRI to confirm the position of each electrode point. The simple procedure is shown in Figure 2.

In addition to SEEG surgically implanted electrodes, SINO can also perform intraoperative cortical electrocorticography, lesion removal and radiofrequency damage surgery. The prospective observational analyses included patients with medically refractory focal epilepsy who underwent robot‐assisted stereotactic placement of depth electrodes for extraoperative brain monitoring. Two of our patients underwent the radiofrequency damage surgery, and other six patients underwent lesionectomy with SINO robot.

### 2.3 Statistical analysis

Statistical analyses were performed using SPSS 20.0 software. The difference between two groups was analyzed using two tailed Student's t test. A value of *p *< .05 was considered a statistically significant difference.

## 3 RESULTS

Operative data for our cohort are reported in Table 1. This study collected 24 SEEG patients admitted to the epilepsy center in neurosurgery department of the Second Hospital of Hebei Medical University from August 2016 to February 2019, including 16 with SINO robot‐assisted SEEG system and eight with frame‐SEEG technology. The age of patients underwent frame SEEG surgery ranges from 10 to 41 years old, and the patients underwent robot‐assisted SEEG surgery in the cohort had a range of 4–38 years of age. The total number of implanted electrodes was 71 and 171 of frame and robot, respectively. Robot‐assisted SEEG neurosurgery had higher accuracy, and the mean error of entry and target point was smaller than that of frame SEEG surgery. No bleeding or infection occurred postoperatively. Electrode displacement was observed in two patients, both the entry points were orbital frontal, one in the frame system and one in the robot assistant system. The average placement time of each electrode in robot assisted system surgery was less than that in frame system surgery. The patients were followed up for 4–12 months, and eight patients recurred in 1 week (one frame, three robots), and three patients recurred in one month (one frame, two robots). Six months after surgery, only one patient who underwent radiofrequency damage had a recurrence.

**TABLE 1:** Comparison of clinical parameters between the two implantation methods

		Implant type				
Variate	Index	Robot (*n* = 16)	Frame (*n* = 8)	Statistical	Value	*p*‐Value
Gender	Male	9 (56.25%)	3 (37.50%)	Fisher's exact test	–	.667
	Female	7 (43.75%)	5 (62.50%)			
Seizure frequency	Once a week or more	8 (50.00%)	3 (37.50%)	Fisher's exact test	–	.679
	Less than once a week	8 (50.00%)	5 (62.50%)			
Age (years)	Mean ± SD	15.69 ± 9.46	26.00 ± 10.82	Student's *t*‐test	−2.402	.025
Course (years)	Median (IQR)	3.50 (0.92,6.50)	8.00 (2.42,13.50)	Wilcoxon two‐sample test	1.196	0.232
Number of electrodes	Mean ± SD	10.13 ± 2.70	9.00 ± 3.51	Student's *t‐*test	0.871	.393
Average operation time (min)	Median (IQR)	7.60 (6.9,8.35)	13.55 (11.50,16.00)	Wilcoxon two‐sample test	3.707	<.001
Entry point error (mm)	Median (IQR)	1.56 (0.00,2.24)	1.50 (0.00,2.25)	Wilcoxon two‐sample test	0.134	.894
Target point error (mm)	Median (IQR)	2.27 (0.79,3.05)	2.02 (1.03,3.04)	Wilcoxon two‐sample test	−0.012	.991

Abbreviations: IQR, interquartile range; SD, standard deviation.

## 4 DISCUSSION

In the present study, our statistical results show that the robot‐assisted neurosurgery system has shorter operating time, smaller entry and target errors and fewer electrode abnormalities, indicating that the robot‐assisted neurosurgery system has higher operability, accuracy and safety. Robot‐assisted neurosurgery system can improve the accuracy of electrode implantation and the efficiency of surgical treatment. We believe that the advantages of surgical robots shown in our statistical data were very considerable, and their extensive clinical application will bring great improvement to the efficiency and effect of neurosurgery.

So far, most stereotactic neurosurgery still relies on the workflow established about half a century ago (Guo et al., 2018). The basic idea of SEEG created is to analyze the clinical symptomatology and periparoxysmal phase EEG data, to understand the anatomic‐electrical‐clinical correlation and to hypothesize the anatomic localization characteristics of epileptic network (Gonzalez‐Martinez et al., 2013). The most important indications that SEEG tested include cortical structures located in the sulcus (including focal cortical dysplasia), deep cortical structures (including insulin‐cap, limbic system) and deep or paraventricular lesions (such as paraventricular ectopic gray matter and hypothalamic hamartoma) (Iida & Otsubo, 2017). Accuracies and safety for SEEG electrode implantations have been confirmed (Cardinale et al., 2013). Due to its own mechanical error and the error caused by the mounting frame, the stereoscopic frame has a bigger error (Starr et al., 2002). Frameless stereotaxy has been proved to be comparable to frame‐based stereotaxy in accuracy, diagnostic yield, morbidity and mortality (Candela‐Cantó et al., 2018). However, it could still be complicated and time‐consuming because of the intensive manual operation required. To further simplify the procedure and enhance accuracy, robotics was introduced. Robots have superior capabilities over humans in certain tasks, especially those that are limited by space, accuracy demanding, intensive and tedious. Clinical benefits have been shown in the recent surge of robot‐assisted SEEG surgical interventions (González‐Martínez et al., 2016; Mcgovern et al., 2018; Mullin et al., 2016; Ollivier et al., 2017; Zeng et al., 2017 ).

The robot‐assisted SEEG system has varieties of advantages, including high accuracy, fewer complications and ease of operation (Sharma et al., 2019; Spyrantis et al., 2018 ). At the same time, each component of the robot‐assisted SEEG system has its own advantages. The manipulator has the characteristics of flexible operation and small system error. The software system has multi‐mode image fusion (including MRI, CT, PET) and reconstruction functions (including vascular reconstruction, 3D reconstruction). Puncture system accessories is complete and easy to operate. In addition, angiography methods, such as MRI, CT, DSA, MRI structural images fusion, and registration methods, including scalp registration points, facial tracking, skull fixation registration points, are also worthy of attention. Medical robot system is composed of auxiliary planning navigation system and auxiliary operating subsystem. Before surgery, doctors can obtain three aspects of information, which are the anatomical structure of the patient's surgical site and adjacent areas, surgical planning and surgical path planning. In addition, simulation operation can also be carried out to understand the location of surgical instruments in the diseased tissue and the surrounding tissue information.

Comprehensive epilepsy center is necessary to establish a multidisciplinary team of SEEG. SEEG electrode implantation assisted by surgical robot system is safe, accurate and mature. The close cooperation between engineers and clinicians, the continuous improvement of technical accessories and the constant update of software system have greatly promoted the development and application of domestic surgical robots. Intelligence and precision are the inevitable trend in the development of neurosurgery.

## CONFLICT OF INTEREST

The authors declare no conflict of interest.

## ETHICS APPROVAL AND CONSENT TO PARTICIPATE

This study was approved by the Ethics Committee of The Second Hospital of Hebei Medical University (No. 2019‐R172)

## AUTHOR CONTRIBUTIONS

Each author has met the BMC Bioinformatics authorship requirements. Di Zhang, Xuehua Cui and Wenling Li conceptualized the study. Di Zhang, Xuehua Cui, Jie Zheng, Shunyao Zhang, Meng Wang and Wenling Li generated, analyzed and interpreted the data. Linxia Sang performed statistical analysis. Di Zhang, Xuehua Cui and Wenling Li wrote and/or revised the manuscript. All authors have read and approved the final manuscript.

### PEER REVIEW

The peer review history for this article is available at https://publons.com/publon/10.1002/brb3.2347

## Data Availability

The datasets during and/or analyzed during the current study are available from the corresponding author on reasonable request.
